# Mapping cycling patterns and trends using Strava Metro data in the city of Johannesburg, South Africa

**DOI:** 10.1016/j.dib.2016.11.002

**Published:** 2016-11-09

**Authors:** Walter Musakwa, Kadibetso M. Selala

**Affiliations:** a University of Johanesburg, Johannesburg, Gauteng, South Africa; b University of Johannesburg, Department of Quality and Operations Management, South Africa

**Keywords:** Strava, Cycling patterns, Johannesburg, Cycling trips, Smart mobility

## Abstract

Plans for smart mobility through cycling are often hampered by lack of information on cycling patterns and trends, particularly in cities of the developing world such as Johannesburg. Similarly, traditional methods of data collection such as bicycle counts are often expensive, cover a limited spatial extent and not up-to-date. Consequently, the dataset presented in this paper illustrates the spatial and temporal coverage of cycling patterns and trends in Johannesburg for the year 2014 derived from the geolocation based mobile application Strava. To the best knowledge of the authors, there is little or no comprehensive dataset that describes cycling patterns in Johannesburg. Perhaps this dataset is a tool that will support evidence based transportation planning and smart mobility.

**Specifications Table**

**Table:** 

Subject area	Urban and Regional Planning
More specific subject area	Transportation planning and Smart Cities
Type of data	Tables, maps and figures
How data was acquired	STRAVA METRO: STRAVA METRO collects data of cycling activities uploaded from the GPS based mobile application STRAVA
Data format	Analysed
Experimental factors	N/A
Experimental features	N/A
Data source location	Johannesburg, South Africa. -26.20 latitude and 28.04 longitude
Data accessibility	Data is with this article

**Value of the data**

• This data is useful because, for the first time the spatial cycling patterns, trends and attributes for the entire extent of Johannesburg area depicted for the year 2014.
• Mapping the cycling patterns and trends from Strava data allows city managers, transportation planners, policy makers and other stakeholders to promote evidence based infrastructure planning (cycling lanes) that will foster smart and sustainable cities.
• The maps and the data provided are useful for other researchers in Johannesburg and South Africa, as well as elsewhere who have struggled to obtain an up-to-date, and holistic dataset on cycling patterns in Johannesburg.
• The maps are useful for identifying cycling hot spots and cold spots as well portraying the correlationship between cycling and income.

## 1 Data

The data presented herein shows the total cycling trips and attributes in Johannesburg for the year 2014 as analysed from Strava Metro. The cycling trips are divided into recreational and commuting trips. Likewise, the temporal variation of total cycling trips is also portrayed. Lastly, the correlationship between cycling and income is portrayed.

### 1.1 Total cycling trips and temporal coverage

The number of cycling trips recorded by Strava Metro for Johannesburg was 84,297 for year 2014. Only 20% of the cycling trips are for commuting whereas recreational trips accounts for approximately 80% of the cycling trips (Table 1).

**Fig. 1:**
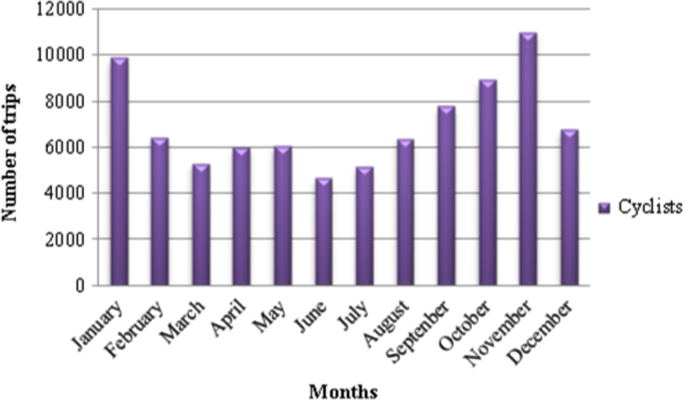
Cycling trips per month in Johannesburg for the year 2014.

**Fig. 2:**
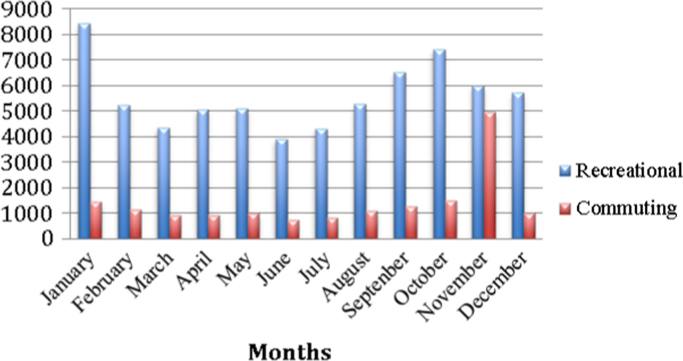
Monthly commuting and recreational trips in Johannesburg for the year 2014.

Similarly, it is also noted that the highest number of cycling trips are in the summer months of September (7786), October (8928), November (10,997), December (6578) and January (9880) (Fig. 1). In the middle of winter (June and July months) the lowest cycling trips of 4660 and 5151 respectively are recorded. This pattern is common for both recreational and commuting cycling patterns (Fig. 2).

**Fig. 3:**
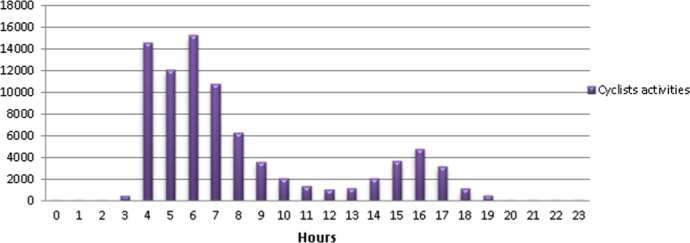
Cycling activities over a 24-hour period in Johannesburg for the year 2014.

**Fig. 4:**
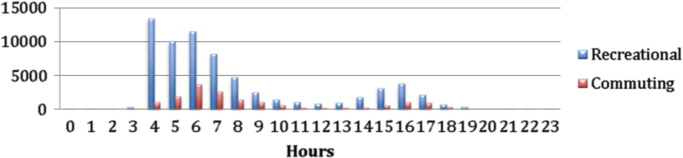
Cycling activities over a 24-hour period for both recreational and commuting trips in Johannesburg for 2014.

Over a twenty four period in 2014, the cycling trips follow a discernable pattern, where most cycling activities peak in the early morning hours between 0400 h and 0900 h and peak again between 1500 h and 1700 h (Fig. 3), for both recreational and commuting trips (Fig. 4). During the day, between 1100 h and 1400 h as the temperature sours and people going about their daily routines cycling activities tend to decline (Fig. 3).

## 2 Spatial coverage

**Fig. 5:**
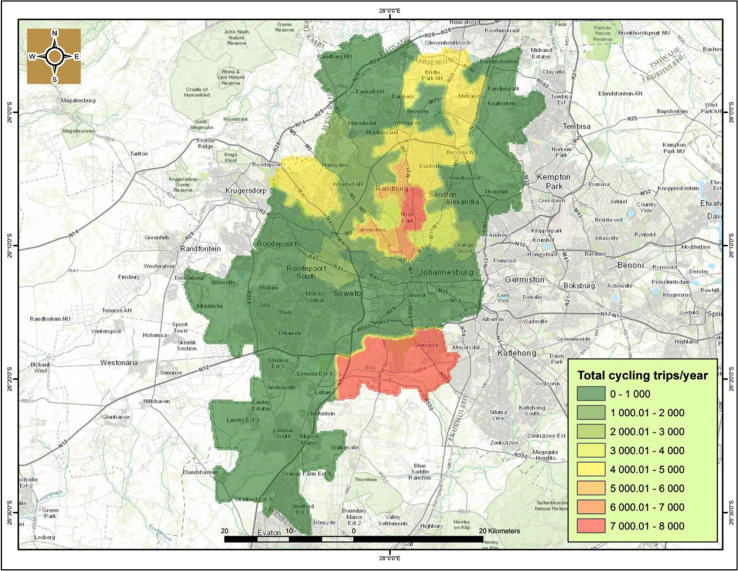
Cycling trips per suburb in Johannesburg for 2014.

The drawback of conventional and traditional methods of data collection techniques on cycling patters such as traffic counts, is that they cover a limited spatial extent and are often cumbersome to conduct [1], [3]. With Strava Metro data this is circumvented, as it covers a broader spatial extent (national, provincial and local) and it is regularly updated. Fig. 5 shows the spatial coverage of cycling activities within the city of Johannesburg in 2014.

**Fig. 6:**
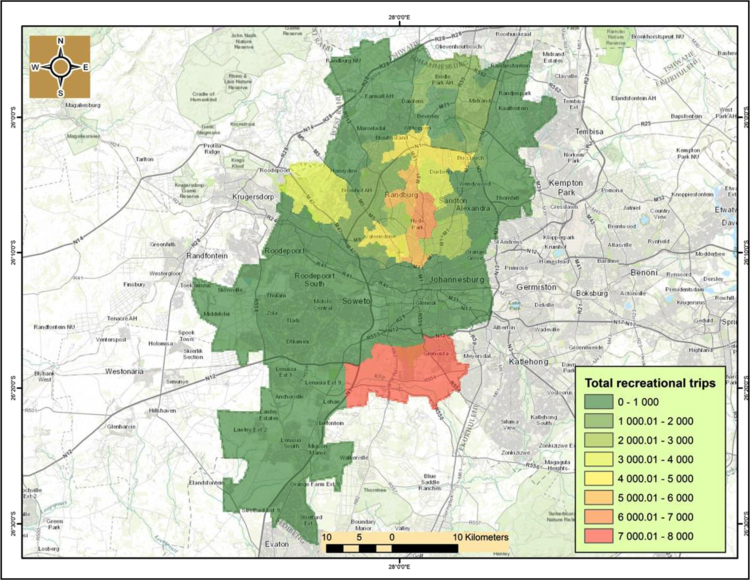
Recreational cycling trips in Johannesburg for 2014.

**Fig. 7:**
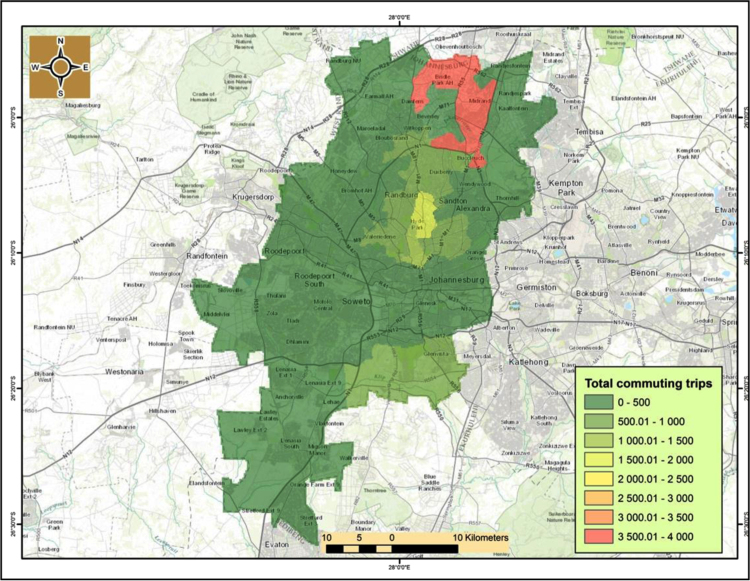
Commuting trips per suburb in Johannesburg for 2014.

It noticeably emerges that the northern and northwest suburbs’ such as Hyde Park, Carlswald, Parkview, Sandton, Midrand, Randburg, and Honeydew contain the highest number of cycling activities of between 3000-to-8000 trips per year in 2014. Hyde Park and adjacent suburbs in Sandton are the hot spots of cycling activities in Johannesburg. This pattern is also largely similar for both recreational and commuting activities (Fig. 6, Fig. 7).

The cycling cold spots with limited or no cycling activity are mostly south of Johannesburg׳s central business district (CBD), with the exception of Kibler Park, south east of Johannesburg that is a cycling hotspot for both recreational and commuting activities. Therefore, this dataset is quite valuable because it can be used to inform evidence based planning and guide infrastructure planning unlike the current scenario where cycling lanes where built in the Johannesburg CBD which is a cycling cold-spot. As a result, other road users such as cars and pedestrians are now using the cycling lanes.

**Fig. 8:**
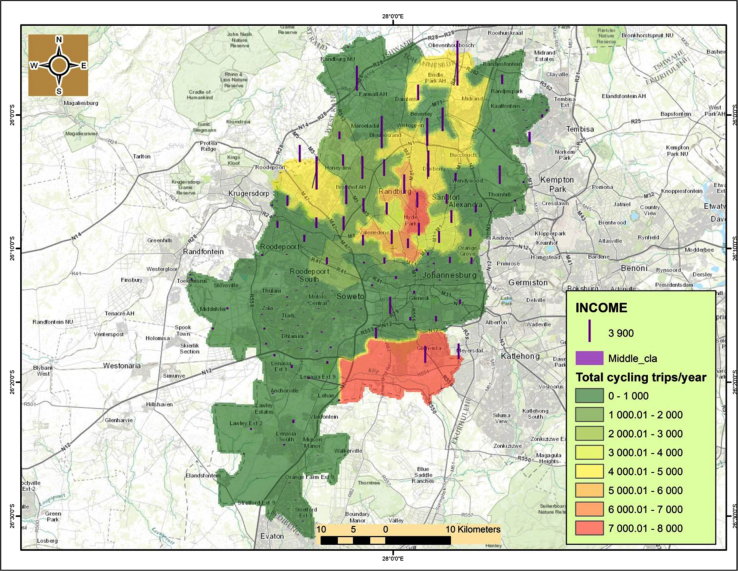
Cycling trips in relation to middle-income households.

**Fig. 9:**
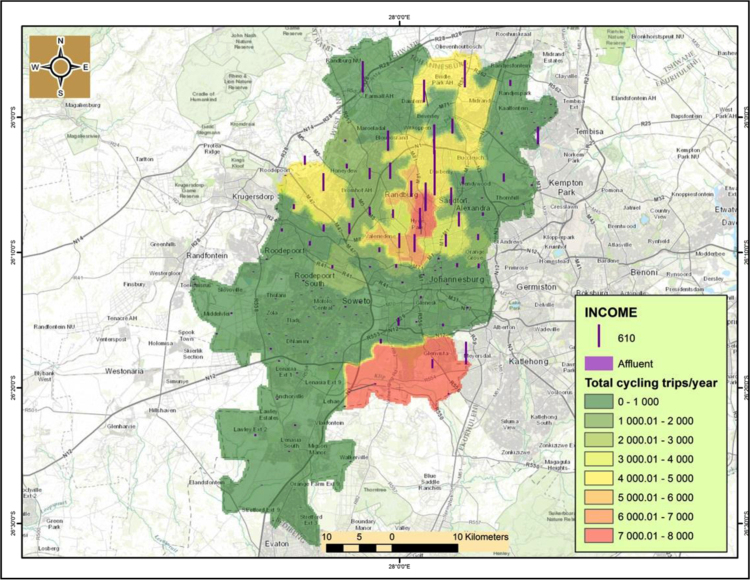
Cycling trips in relation to affluent households.

**Fig. 10:**
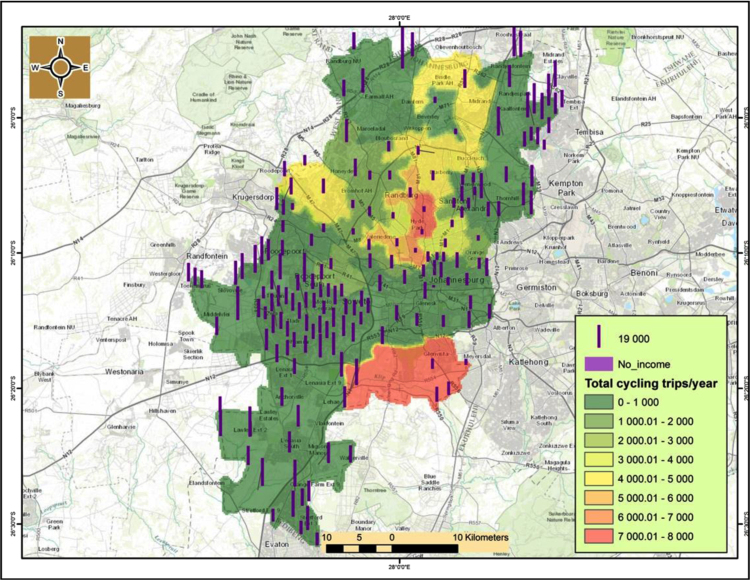
Cycling trips in relation to households with no income in Johannesburg.

An interesting observation is that there is a correlationship between cycling and household income.^1^ From Fig. 8, Fig. 9 it is evident that cycling uptake is mostly for the middle class and the affluent that mostly reside in the northern suburbs of Johannesburg CBD such as Sandton. Cycling is not being used regularly by those with little or no income particularly in the southern suburbs such as Soweto and Orange farm as well as north-western suburbs such as Alexandria (Fig. 10).

## 3 Materials and methods

Data from Strava Metro was obtained from Strava for Johannesburg for the year 2014. Strava Metro utilizes data from the Strava mobile application, which is a global positioning system (GPS) enabled smartphone application that tracks bicycle rides and uploads the data to an online community of other users [5], [6]. Millions of people upload their cycling trips to Strava every week via their smartphone or GPS device. Strava Metro anonymizes and aggregates this big data and packages this data in geographic information systems (GIS) format to enable cities to better understand cycling patterns [1]. Strava Metro has three licenses of this data, namely, (1) streets, (2) nodes and (3) origins and destination licenses. Currently we were unable to acquire the streets or nodes license that give better insights into cycling patterns [5], [6]. Accordingly in May 2015, we purchased the origins and destination license that records the origin and destination of cycling activities. Nevertheless, currently the city of Johannesburg does not possess any information on cycling patterns. Hence this dataset would be an appropriate start into providing such information that notes cycling patterns and behaviors [2].

Data purchased from STRAVA was in database (dbf), Microsoft Excel and shape file (shp) format. The dbf and Microsoft Excel contained all the cycling attributes whilst the shape file contained the location (suburbs) of where the cycling activities took place in Johannesburg. Accordingly, these files where joined using ArcGIS 10.3 software so as to spatially analyse the data. Cycling patterns where analysed on the basis of the type (recreational or commuting), temporal and spatial coverage. The analysis was also at city and neighbourhood level. Geospatial modelling software (GME) as well as the spatial analyst and map algebra functions of ArcGIS software were utilized to calculate the descriptive statistics (median) of cycling patterns. The median was chosen because of the extreme scores in the data. Income data from the 2011 census was acquired from Statistics South Africa to correlate cycling patterns and quality of life. Visualization of the analysis was done using ArcGIS software.

Lastly, although Strava data assists in showing cycling patterns, there is often missing data, since not everyone uses Strava to record cycling activities. Nevertheless, to the best knowledge of the authors this datasets is the most comprehensive cycling dataset that covers the entire Johannesburg [4].

## Figures and Tables

**Table 1:** Cycling activities for the year 2014 in Johannesburg.

**Activity**	**Trips**	**%**
Commuting	16,844	20%
Recreational	67,453	80%
Total	84,297	100%

## Footnotes

1 The classifications of no income, middle-income households and affluent households derived from: Bureau of Market Research (2011). Household Income and expenditure patterns in South Africa 2011. Pretoria: University of South Africa.
